# Cross-cultural adaptation of the medical engagement scale into Danish general practice setting

**DOI:** 10.1080/02813432.2019.1608042

**Published:** 2019-05-06

**Authors:** Peder Ahnfeldt-Mollerup, Helle Riisgaard, Jens Søndergaard, Jørgen Nexøe

**Affiliations:** Research Unit of General Practice, Department of Public Health, University of Southern Denmark, Odense, Denmark

**Keywords:** Family practice, Health service research, Leadership behaviour, Translation, Work engagement

## Abstract

**Introduction:** The need to involve doctors in healthcare leadership has long been recognized by clinical staff and policymakers. A Medical Engagement Scale has been designed in England to evaluate levels of medical engagement in leadership and management.

**Objective:** The aim of this study was to translate and adapt the scale and to test the translated version for comprehension and suitability in Danish general practice setting.

**Design and method:** The process involved forward translation, backward translation, and field tests. The field tests included cognitive debriefing interviews. In all 30 general practitioners and 5 non-general practitioners participated in the process of translation. After using the scale among 1652 general practitioners statistical analysis was carried out to test internal consistency.

**Setting:** The study was carried out in general practice in Denmark.

**Results:** Several changes made during the process in order to achieve a Danish version that is acceptable, understandable and still capable of measuring medical engagement comparable of the original English version. Analysis of scale internal consistency using Cronbach’s alpha revealed acceptable reliability for all three meta-scales, which ranged from 0.69 to 0.81. The overall tool achieved a Cronbach’s alpha of 0.89.

**Conclusion:** The Danish version of the Medical Engagement Scale is a valid and reliable tool that is acceptable and relevant for general practice in Denmark.

**Key points**
- This study describes the cross-cultural adaptation of the Medical Engagement Scale from a UK primary healthcare setting to a Danish primary healthcare setting.
- The process produced a relevant and acceptable questionnaire measuring medical engagement.
- Internal consistency revealed acceptable reliability
- The translation of the scale provides the possibility to use this scale for practical and academic purposes.

## Introduction

The value of medical doctors engaging in leadership roles forms the organisation in which they work and gives rise to the concept of medical engagement in leadership and management [1]. Medical engagement is defined as, “*the active and positive contribution of doctors within their normal working roles to maintaining and enhancing the performance of the organisation which itself recognises this commitment in supporting and encouraging high-quality care*” [1]. To be able to operationalize medical engagement in leadership and management a ‘Medical Engagement Scale’ was developed in Birmingham, UK [1]. The Medical Engagement Scale was developed in collaboration between The Enhancing Engagement in Medical Leadership project team in the UK and an external company, Applied Research Ltd. [1]. The scale is easy and relatively unobtrusive to complete, and can give useful information about both the personal feelings of medical doctors and the organisational culture [2]. The information from the Medical Engagement Scale is meant for providing recommendations of organisational strategies in order to enhance medical engagement and performance at work [1,3]. However, recommendations from the initial development of the Medical Engagement Scale was that further development work was required to for the scale to be appropriate for general practitioners (GPs) working in primary care settings [1]. Thus, a separate version of the Medical Engagement Scale has been developed for the primary care setting. This scale reflects the competencies needed for engagement in general practice and primary care trusts in UK [4,5]. The Medical Engagement Scale for Primary care was tested in two separate Primary Care Trusts in UK. The Medical Engagement Scale for Primary Care was shown to be sensitive enough to differentiate not only between the levels of medical engagement of doctors in Primary Care Trusts, but also between the consistency of individual ratings across the three organisational perspectives rated in the scale. [6].

The Medical Engagement Scale for Primary Care has 54 items and consists of nine facets that are grouped into six meta-scales. The meta-scales are divided into three (process) dimensions: 1) Employing knowledge, 2) Connecting emotionally, and 3) Pursuing achievement and another three (outcome) dimensions: A) Personal contribution, B) Job support, and C) Joint working [7]. The three process dimensions and the three outcome dimensions create nine facets. Each facet constitutes a separate component of medical engagement. The frame for Medical Engagement Scale for Primary Care is shown in Box 1.

The primary care setting in Denmark is similar to the UK primary healthcare setting. In both countries most GPs are private entities with one or more GPs in each practice [8]. The GPs work as gatekeepers for the healthcare systems and carry out the same services. To a large extent GPs are organised in the same kind of practices with staff employed to support the GPs. Consequently, it was assumed that the Medical Engagement Scale could be transferred by a cross-cultural adaptation from an English into a Danish general practice setting.

The purpose of this study was:

- *To translate and adapt the Medical Engagement Scale into Danish and test the translation for comprehension*
- *To test the suitability and especially the content coverage of the Danish version in a GP setting*

## Materials and methods

### Cross-cultural adaptation

The purpose of a translation of the questionnaire was not to achieve a literal translation, but to maintain the essence and the idea of the content in the original questionnaire [9]. This requires both a translation and a cross-cultural adaptation from one language to another and from one culture to another. It has to be checked so that the translated questionnaire is not misunderstood, and whether the target group finds the content of the questionnaire relevant [10,11].

**Figure 1.**
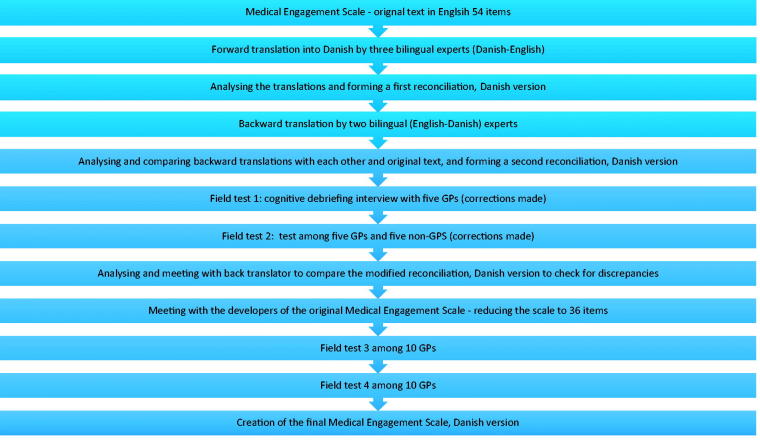
Flow diagram of the cross-cultural adaptation of the Medical Engagement Scale from English into a Danish general practice setting.

Before the translation process started a few comments from the original questionnaire were discussed with the developers, and then the questionnaire was revised. The translation process largely followed the guidelines outlined in the literature [12–15]. The cross-cultural adaptation was undertaken in collaboration with the developers of the original Medical Engagement Scale. The process of cross-cultural adaptation of the scale involved several stages (Figure 1).

### Forward translation

The first forward translation was produced by a “bilingual expert panel” fluent both in the target and source languages. This panel represented the target audience of the questionnaire in terms of age and gender. Three native Danish speakers, working independently, translated the original 54 items of the Medical Engagement Scale into Danish. Two of the translators were general practitioners with knowledge of primary healthcare in Denmark, and the third translator was a Bachelor of Arts and Communication employed as a researcher in the Research Unit of General Practice. To assist the translation, English-Danish and English Dictionaries (Gyldendals Røde Ordbøger) and Oxford Advanced Dictionary, respectively, were used.

### Analysing the forward translation

When each forward translation was complete, the three translators met to discuss discrepancies between their versions. The three translations were quite similar. There were, however, a few items that included words or concepts that could be translated in different ways depending on the intended meaning of the original English text, (e.g. ‘going the extra mile’, ‘allegiance’, and ‘individual assessment’). Consensus was reached for each case representing the idea of the question rather than just translating the word directly into Danish. The bilingual panel primarily emphasised the production of conceptually equivalent translations, whereas linguistic equivalence was considered of secondary importance. The bilingual expert panel produced a first ‘reconciliation’ Danish version, which they considered to be the best translation of the original English text.

### Backward translation

The first reconciliation of the Danish version was translated back into English independently by two different bilingual English-Danish speakers. One of the translators was a native English (American) speaking medical doctor working in Denmark and the other translator was a native Danish speaking person, who had spent several years in English speaking countries. Both had vast experience with translating questionnaires. When each backward translation was complete one of the back translators met with one person representing the ‘bilingual expert panel’. The backward translations were compared, and the translation process (both forward as well as backward translations) was discussed, and differences between these and the original one were analysed to check whether any information was lost in translation. The two backward translations were quite similar to each other and to the original English version. Many of the differences were minor and simply arose from different ways of saying the same things – maybe some of the minor differences arose simply because of linguistic differences. Corrections were made in the Danish translation.

### Field test 1 - Cognitive debriefing interviews

A field test with cognitive debriefing interviews was carried out to ensure acceptance among GPs of the Danish translation of the Medical Engagement Scale and to ensure face, content and criterion validity, which is to test the extent to which the questionnaire is subjectively viewed as covering the concept it purports to measure, and to ensure that each item refers to the original questionnaire. It thus refers to the transparency or relevance of the questionnaire as it appears to respondents. The pilot study was carried out as cognitive debriefing interviews. Face-to-face interviews were conducted with five GPs bilingual in Danish and English. The interview schedule included three steps. The initial step was that the respondents were asked to complete the questionnaire in the presence of the interviewer but as if he or she were on his/her own. They were discouraged from asking questions before they had finished completing the questionnaire. After completion, they were asked standardized questions on understandability, acceptability and comprehensiveness of each item plus any additional questions that might have emerged during the translation process [9]. The interviewer observed and noted any obvious problems during the process of completing the questionnaire. Questions were then asked about any problems observed and the respondents’ opinion of the questionnaire. In particular they were asked:

- *“Whether the questions were relevant, easy to understand and acceptable”*
- *“If they found any of the questions irrelevant, ambiguous or inappropriate”*
- *“Whether they thought that any important aspect of their experience had been omitted”*
- *“Any specific questions from the translation panel such as how specific items were understood or regarding preferences for alternative wordings”.* [9]

Finally, the respondents were asked to compare each item with the original English one to see if they would have answered differently, if they had read the English item instead of the Danish one, and if they could come up with a different and better translation than the original one. Words that the first ‘bilingual panel’ were searching for, but were unable to find, could be identified. After the cognitive debriefing interviews, it was clear that most of the questions were understandable, acceptable and comprehensive, and there was agreement among the respondents on which questions were problematic and could be left out. The questions to be left out was primarily due to the fact that these questions did not apply much to a GP setting or the questions could not be answered for both single-handed GP and GPs from partnership practices.

### Field test 2

To increase the number of responses and to make sure that as many comments as possible were taken into account, a second field test was performed to cross-check for content and criterion validity. Five GPs and five non-GPs (one Master of Arts in Business Communication, one Master of Arts in Communications, a chiropractor, and a psychologist) received the questionnaire. Two of the GPs were highly skilled writers (novelists) with extensive knowledge of the Danish language. All of them were asked to complete the questionnaire and comment on each item, and they were further asked to compare the Danish version against the English original and to come up with an alternative translation if relevant. This field test basically identified the same problems as the ones seen during the cognitive debriefing interviews. However, some items were suggested rephrased into more accurate and less academic Danish.

### Analysing data to create the second reconciliation of the scale

Having completed the cognitive debriefing interviews and the field test an additional meeting was held with one of the back-translators to ensure that the suggested rephrasing did not change the meaning of the original question. A version of the questionnaire with the comments from the field test omission of the items that were suggested to be left out was completed. This version was then sent to the developers of the Medical Engagement Scale for comments before the second reconciliation of the scale was completed.

### Meeting with the developers

The second reconciliation of the scale was completed after a meeting with the developers of the original questionnaire in Birmingham in the UK. They stressed that the Medical Engagement Scale was to measure a ‘willingness’ to do different tasks and not to measure the ‘actual behaviour’. The questionnaire is a psychometric tool measuring the GP’s inner feelings or willingness regarding the specific questions. The developers stressed the importance of this issue. Furthermore, each question and the nine facets were to be strictly identifiable measuring the same concept as the English version, but could be formulated in a different way. The Medical Engagement Scale for Primary Care could thus be called “The Danish Version” to distinguish this one from the English one. The purpose of this was to highlight the fact that, though the two versions of the Medical Engagement Scale are actually measuring the same concept and are comparable, there are actually two versions and not one, and that the survey is made in another context/cultural setting. Each question in the Danish version of the questionnaire was then reanalysed, and as single-handed GPs were to be able to answer the questionnaire as well, the questions that were not applicable for single-handed practices were identified. Rephrasing was then made.

### Field test 3 + 4

Two further field tests were carried out among single-handed GPs, GPs in partnership practices and GPs in collaboration. All questions were assessed by both GPs from single-handed practices and partnership practices to be relevant, appropriate and acceptable. This reduced the total number of questions from the original 54 items to 36, with four items in each of the nine facets. Minor corrections were made after the third pilot study, which is why an additional forth pilot study was carried out. After this fourth and final pilot study, no further corrections or rephrasing were made.

### Measuring medical engagement in the general practice setting

The questionnaire was distributed by email April 2013 to all GPs in Denmark. A total of 1652 GPs completed the questionnaire. The results from this survey has been published elsewhere [7].

The Medical Engagement questions were presented as a five-point Likert scale (strongly agree to strongly disagree). For each question, the answer was given a number from 1–5 for positive answers and from 5–1 for negative answers. Questions had to be answered in sequence and each question had to be answered before proceeding on to the next question.

### Analysing responses from the survey

The outcomes of this study were the ratings in the form of an individual index of the overall medical engagement for each GP, the six meta-scales and the nine facets. All items in the scale had same value. The rating of the overall medical engagement index was calculated as the mean of all the ratings. In order to ensure statistical comparability across the scales, the medical engagement scales were transformed into z-scores (standardized scores) rather than raw scores.

## Results

The cross-cultural adaptation of the Medical Engagement Scale into a Danish general practice was carried out and the number of items was reduced from 54 to 36.

Analysis of scale internal consistency using Cronbach’s alpha revealed acceptable reliability for all three meta-scales, which ranged from 0.69 to 0.81 (Table 1). The overall tool achieved a Cronbach’s alpha of 0.89. Correlations between meta-scales means were statistically significant (*p* < .001) and positive. Structured equation modelling analyses revealed the coefficient of determination is quite high with a value of 0.954 indicating, that the used model fits well for the questionnaire. However, an upper bound Root Mean Square Error of Approximation below 0.08 is the target, where we have the results for total: 0.081, singlehanded: 0.092 and partner: 0.080. Though these do not reach the target we consider the results as acceptable. The Medical Engagement has a three-by-three-dimensional structure and these dimensions are closely related.

**Table 1.** Statistical analyses of the 36 items in the Medical Engagement Scale (1,652 respondents) for each item and meta-scales.

Crohnbachs alfa	
MES-total	0.8969
Meta-scales	
1) Employing knowledge	0.7591
2) Connecting emotionally	0.7426
3) Pursuing achievement	0.8201
A) Personal contribution	0.6945
B) Job support	0.8067
C) Joint working	0.7634
Structured equation modelling	
Coefficient of determination	0.954
Upper bound Root Mean Square Error of Approximation:	
Total	0.081*
Singlehanded practices	0.092*
Partnership practices	0.080*

*The target is a result below 0.080.

## Discussion

The Medical Engagement Scale for primary care was translated and adapted for a Danish context. The number of questions was reduced from 54 to 36. As the same conditions apply for Danish GPs as for GPs in UK, the scale could with minor adaptions be transferred from a UK context to a Danish context. The final version of the Danish Medical Engagement Scale measures the same concept, as the original English one, but the translation, the adaption and reduction in number of questions are limitations to directly comparing data across languages and cultures, and this has to be taken into account when making comparison with surveys based on the English version.

However, the purpose of the translation was not to achieve a literal translation, but instead to maintain the essence and the idea of the content in the original questionnaire. In the process of the cross-cultural adaptation, four field tests were carried out to ensure a valid questionnaire, which was acceptable among GPs and relevant for the GP sector, and furthermore, easy to understand, administer and respond to.

To adapt questionnaires from one language to another and from one culture or setting to another will affect the result despite the accuracy of the adaptation. Though the original and the Danish version of the Medical Engagement Scale are measuring the same concept, there are actually two versions and not one, and comparing results has to be made carefully. The strength of this study was the several stages of translation, working in collaboration with the developers of the original scale and the field tests that were carried out in an applicable population. Statistical analyses revealed acceptable results.

Employee or work engagement can be (and has been) measured in different ways. For example, the Utrecht Work Engagement Scale [16], which are conceptually derived from the converse of the three components of occupational ‘burnout’ [17], and consequently some authors state that employee or work engagement is the ‘positive antithesis’ of burnout [18]. In healthcare settings in the literature, there are different measures of engagement that have been studied in a recent review [19], where the different measures of engagement can be categorised into three headings: a psychological state of mind; a blended construct of attitude and behaviour; and relationships between staff members. The Medical Engagement Scale adds another dimension to engagement, namely the support from the organisation.

### Implications

The Danish version of the Medical Engagement Scale can be used for evaluating medical engagement in general practice to direct initiatives to where improvements are needed to promote engagement. The Medical Engagement Scales identifies specific areas of interest such as personal contribution from the individual GP, organisational support perceived by the GP and collaboration within the organisation, all of which are important issues for the healthcare service provided by GPs [20–23]. Applied Research Ltd. has a database, where anonymous data is collected from each study using the Medical Engagement Scale, also form the primary care version. On each occasion including data from this study they include "marker " items from the main MES so they can relate a new model to the existing norms. Studies on causality between the findings using the Medical Engagement Scale and associations with factors of importance in the healthcare sector could be relevant and is likely to be able to influence the continuous development of this area of research.

## Conclusion

The Medical Engagement Scale for primary care was successfully translated and adapted for a Danish context and seems relevant, appropriate and acceptable for GPs in both single-handed and partnership practices.
